# Overview of Artificial Intelligence in Breast Cancer Medical Imaging

**DOI:** 10.3390/jcm12020419

**Published:** 2023-01-04

**Authors:** Dan Zheng, Xiujing He, Jing Jing

**Affiliations:** Laboratory of Integrative Medicine, Clinical Research Center for Breast, State Key Laboratory of Biotherapy, West China Hospital, Sichuan University and Collaborative Innovation Center, Chengdu 610041, China

**Keywords:** artificial intelligence, breast cancer, radiomics, radiogenomics, medical imaging

## Abstract

The heavy global burden and mortality of breast cancer emphasize the importance of early diagnosis and treatment. Imaging detection is one of the main tools used in clinical practice for screening, diagnosis, and treatment efficacy evaluation, and can visualize changes in tumor size and texture before and after treatment. The overwhelming number of images, which lead to a heavy workload for radiologists and a sluggish reporting period, suggests the need for computer-aid detection techniques and platform. In addition, complex and changeable image features, heterogeneous quality of images, and inconsistent interpretation by different radiologists and medical institutions constitute the primary difficulties in breast cancer screening and imaging diagnosis. The advancement of imaging-based artificial intelligence (AI)-assisted tumor diagnosis is an ideal strategy for improving imaging diagnosis efficient and accuracy. By learning from image data input and constructing algorithm models, AI is able to recognize, segment, and diagnose tumor lesion automatically, showing promising application prospects. Furthermore, the rapid advancement of “omics” promotes a deeper and more comprehensive recognition of the nature of cancer. The fascinating relationship between tumor image and molecular characteristics has attracted attention to the radiomic and radiogenomics, which allow us to perform analysis and detection on the molecular level with no need for invasive operations. In this review, we integrate the current developments in AI-assisted imaging diagnosis and discuss the advances of AI-based breast cancer precise diagnosis from a clinical point of view. Although AI-assisted imaging breast cancer screening and detection is an emerging field and draws much attention, the clinical application of AI in tumor lesion recognition, segmentation, and diagnosis is still limited to research or in limited patients’ cohort. Randomized clinical trials based on large and high-quality cohort are lacking. This review aims to describe the progress of the imaging-based AI application in breast cancer screening and diagnosis for clinicians.

## 1. Introduction

By 2020, breast cancer surpassed lung cancer as the most common type of cancer worldwide, with the highest incidence and second highest mortality rate [1]. With the continuous development of new drugs with better efficacy and lower toxicity, breast cancer has become a malignant tumor with a better prognosis, and the concept of prevention and treatment of breast cancer has gradually shifted to treating it as a chronic disease and formulating a series of management strategies from screening to treatment and follow-up after diagnosis. Therefore, early detection of breast cancer has become the key link in the whole management of breast cancer, which can help improve the cure rate and significantly reduce the mortality rate [2,3].

Imaging detections including mammography, ultrasound, magnetic resonance imaging (MRI), and positron emission tomography (PET) are the most essential image tools for breast cancer auxiliary diagnosis [4]. Mammograms are mainly used in breast cancer screening in asymptomatic women due to its good performance in detecting small tumors [5,6,7] using a low-dose of X-ray [8]. It is the only imaging tool that has been proven to decrease breast cancer-related mortality, but it is accompanied by a significantly higher proportion of over-diagnosis [9]. For diagnosed or suspected breast cancer primary loci, ultrasound is the most common imaging detection for tumor staging and biopsy guiding in clinical routine. Compared to mammograms, ultrasound is relatively more versatile, portable, and cost-effective [10], but highly dependent on the well-trained operator. MRI has higher sensitivity than the above two techniques in breast cancer detection, though it is the most expensive one, with low specificity [11,12]. In addition, conflicting evidence obscures the value of MRI in breast cancer [10], limiting its use to high-risk diseases or indiscernible lesions that are difficult to detect by conventional imaging tools such as mammography and ultrasound [12,13]. As another widely used imaging examination, PET can depict the metabolic characteristics of breast cancer as opposed to just anatomic appearance. Moreover, several hybrid imaging techniques have been introduced into routine clinical practice, such as PET/MRI and PET/computed tomography (PET/CT) imaging, facilitating breast cancer diagnosis and staging.

To date, imaging detections have been widely applied in the early detection and clinical staging of breast cancer, however, several tricky issues have become increasingly prominent in clinical practice. On the one hand, the large amount of imaging data generated during the diagnosis of breast cancer places heavy workload on radiologists. On the other hand, images with low quality or ambiguous features limit the diagnostic accuracy of radiologists, and the presentation of subtle or complex disease manifestations may require both imaging and clinical information to make comprehensive judgement [14]. Computer-aid diagnosis (CAD) provides efficient automated image recognition, lesion segmentation, and diagnosis, potentially reducing the workload of radiologists, and improving diagnostic accuracy. With the advances in CAD, more flexible and versatile analyses are constantly emerging, especially image-based artificial intelligence (AI) techniques, significantly improving the clinical value of CAD in breast cancer. To improve and ensure the accuracy of diagnosis, a reliable CAD method with a high-performance computer technique is essential, which affects the interpretation accuracy directly [15]. Therefore, optimizing the performance of AI-based breast cancer screening and diagnosis is of great importance in better assisting the work of radiologists. In this review, we discuss the application of AI-based imaging detections including mammography, ultrasound, MRI, and PET in breast cancer screening and diagnosis.

## 2. Materials and Methods

In this review, we collected studies and reviews concerning the computer-aided diagnosis of breast cancer which were published from the beginning of 2000 to the present on PubMed, medRxiv, Google Scholar, and Scopus database. The key words used for the literature search were as follows: “breast cancer”, “artificial intelligence”, “deep learning”, “machine learning”, “imaging”, “mammogram or mammography”, “ultrasound”, “MRI”, “PET”, “radiomics”, “radiogenomics”. The search was performed using Boolean “AND” and “OR” operators between the main term and keywords. Articles and reviews were manually rejected if they were irrelevant to primary breast cancer diagnosis or specific to the computer-aided diagnosis based on pathological information. Research articles or reviews published in languages other than English were excluded. According to the image detection methods, the literatures were categorized into mammogram-based, ultrasound-based, MRI-based, and PET-based. We did not discuss the role of computer tomography in breast cancer diagnosis because it is less commonly used in breast cancer diagnosis and there is little relevant research.

In Section 2, we introduce the basic conceptions relevant to AI, machine learning (ML), and deep learning (DL). In Section 3, we discuss the characteristics of different image detection methods and their applications in breast cancer diagnosis.

## 3. Results

**Figure 1:**
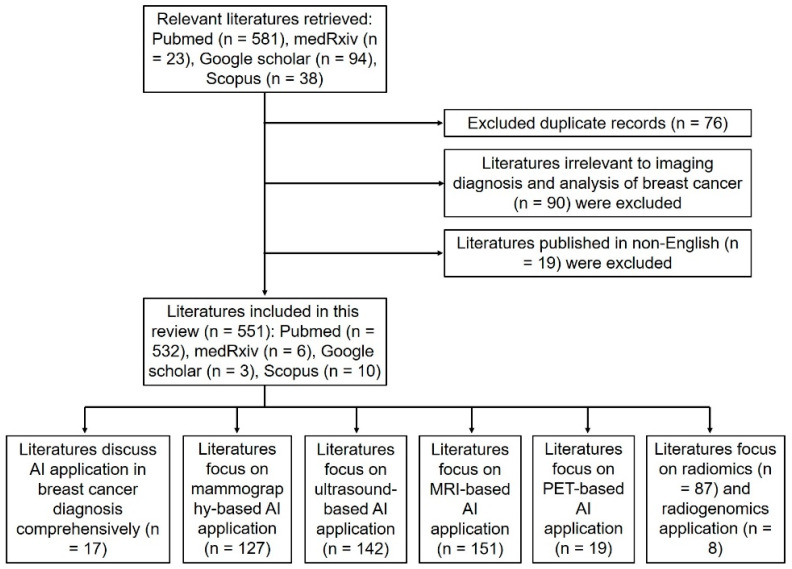
Flowchart to indicate the literature screening process.

A total of 551 studies were identified, and all qualified studies were classified according to the main content covered by each of them. Specifically, studies were grouped into mammography-based AI application, ultrasound-based AI application, MRI-based AI application, PET-based AI application, and radiomics/radiogenomics application in breast cancer, and each of these themes is discussed in Section 3.2.1, Section 3.2.2, Section 3.2.3, Section 3.2.4 and Section 3.2.5. The working process of literature retrieval is presented in Figure 1.

### 3.1. Artificial Intelligence: From Machine Learning to Deep Learning

AI is defined as the ability of a computer to learn algorithms to reason and perform tasks including reading, writing, interacting, problem-solving, and decision-making. ML, a subfield of AI, is mainly used to extract features from training set data and build analytical mathematical models for prediction and analysis of unknown data. The primary model of ML can be divided into the predictive models and the explanatory models, which aim to solve tasks of judging unknown data sets, and describing and explaining the features of unknown data sets according to the purpose of the model built, respectively. When classified by the algorithms, ML can be grouped into supervised algorithms, unsupervised algorithms, and reinforcement algorithms [16]. The unsupervised learning adopts data in the absence of labels and performs tasks about classification (e.g., decision trees, K-nearest neighbors, and support vector machine, SVM) and regression (e.g., linear/non-linear regression, local regression, and ordinary least squares regression), while the supervised learning received labeled data to work on cluster analysis (e.g., hierarchical clustering and K-means clustering) and dimension reduction (liner discriminant analysis and principal component analysis) [17]. The reinforcement learning allows the computer to self-train based on the output consequences of interaction with the environment or the success of the decision, and optimize the decision results by continuously adjusting the algorithm parameters.

Compared with ML, DL also depends on the input information or datasets to acquire decision-making ability, but it does not rely on handcrafted features, and the way it learns is more inclined to the human learning approach. Inspired by the biological nervous system, DL depends on numerous highly interconnected computer units that mimic the neurons of the human brain. These units are constituted of layers, and each layer is fully connected to form the artificial neural networks. The algorithms of DL for image recognition analysis mainly include convolutional neural networks (CNN), deep convolutional neural networks (DCNN), fully convolutional networks (FCN), recurrent neural networks (RNN), and generative adversarial networks (GAN), etc. [18]. In the process of DL, the final goal is decomposed into a series of simple nested mappings (i.e., the concept of layers) and performs a multitude of logical decision-like tasks layer by layer to obtain the answer to the final question. The data input (image data) is presented in the “visible layer”, while the middle convolutional layer performs multiple feature extraction operations on the image. Each convolutional layer contains a large number of convolutional kernels that extract a large number of image features at different locations of the input image for subsequent analysis, which are hidden from the visible data. The dimension of the image feature from the convolutional layer is reduced in the pooling layer and low-resolution but highly representative features of the image are outputted.

**Figure 2:**
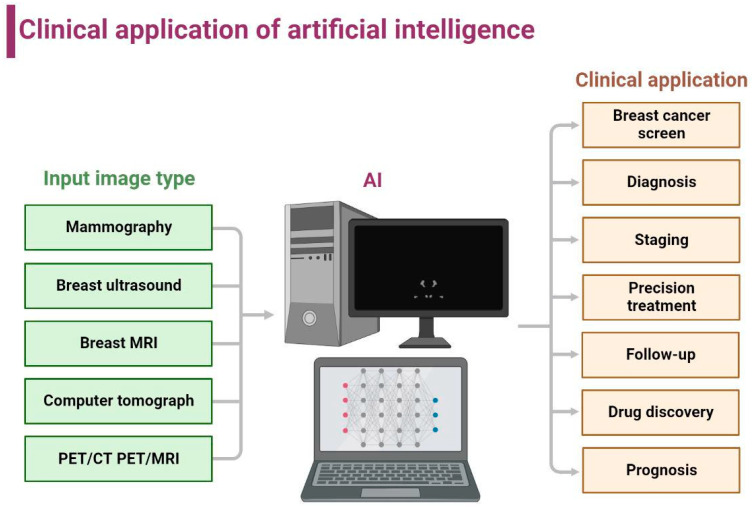
Application of artificial intelligence in breast cancer diagnosis.

ML predictive models are better at explaining predictions than DL because they are built based on well-labeled training datasets. However, in many areas where AI is currently applied, a number of tasks cannot be generalized by mathematical models for interpretation, such as tumor imaging or pathological tissue characterization, and the radiologist can make diagnoses based on their knowledge and experience. DL, which is more similar to the human mindset, can make more factual judgments or predictions “end-to-end” through uninterpretable neural network analysis decision methods. With the parallel high-speed development of computer hardware and data storage flux, AI has excelled in its ability to recognize and learn from high throughput of data and information, a characteristic that extends its applicability to all aspects of cancer research and medicine, such as automatic and accurate detection of cancer from stained tumor slides or radiology images, thus reducing the duplication of work for radiologists and pathologists. AI in the breast cancer field covers a wide range of applications from tumor screening, diagnosis, staging, treatment, follow-up, and drug development [19] (Figure 2).

### 3.2. Application of AI in Breast Cancer Imaging Diagnosis

Breast cancer prevention and control strategies are currently focused on secondary prevention, i.e., enhancing screening for high-risk populations, and early diagnosis is a key component of breast cancer control strategy [20,21]. The greatest value of AI in breast cancer screening may lie in the efficient search for tumor lesions from the huge number of images of healthy people, which greatly reduces the workload of imaging physicians [22]. The development of AI-assisted breast imaging diagnosis is based on computer-aid detection/diagnosis (CADe/CADx) systems. As a primary version of ML, CAD is used to help radiologists with tiny tumor lesions that may be missed by integrating mathematics, statistics, image processing, and analysis by computer. However, the high false positive rate (FPR) and biopsy rate resulting from CAD identification in clinical practice limits its usage in clinical practice [23,24,25]. To improve the performance of CAD, the input image data is used as a training set to build a model, and end-to-end learning is achieved including the processes of clinical data set acquisition, neural network normalization data set processing, ML classification algorithm selection, and overall system performance evaluation [26]. Building a DL-based AI application tool for breast cancer diagnosis requires a large number of high-quality breast examination images as a training dataset; and building DL algorithm that is consistent across people, devices, and modalities [27]. Tumor images can be annotated by outlining the lesion and annotating the lesion features manually or with DL. Manually annotating relies on the knowledge and experience of the imaging specialist, and the lesions outlined manually are used as reference standards for automatic segmentation. However, lesions with small volumes or obscure features are difficult to distinguish from the surrounding normal breast tissue. Moreover, Asian women, especially premenopausal women or patients with received estrogen-replacement therapy may have endo breast [28], making the tumor segmentation more difficult. Therefore, the expert-defined lesions do not fully and accurately represent the true lesion area and extent, and the repeatability consistency is weak [29,30,31].

**Figure 3:**
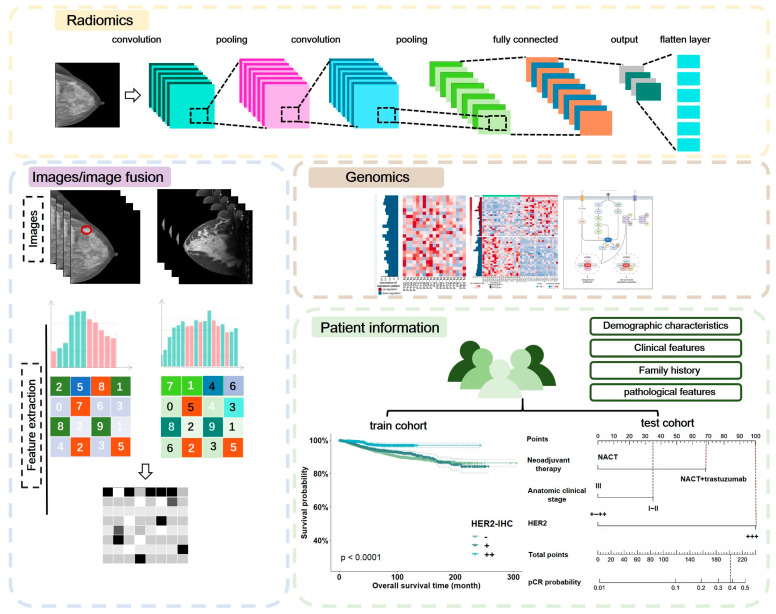
Working model of medical imaging artificial intelligence. NACT, neoadjuvant chemotherapy.

Automatic image segmentation methods generally include thresholding-based, region-based, edge-based, clustering-based, or other segmentation methods, while no accepted gold standard for image segmentation methods has been identified. DL algorithms usually refer to neural network algorithms, and CNN is one of the most developed DL algorithms with convolutional, nonlinear, pooling, and fully connected layers in the computing path (Figure 3). AI applied to breast cancer screening can help reduce or prevent some visible lesions from being overlooked or misinterpreted [32]. However, AI image interpretation tools based on DL are actually most commonly used for secondary review of negative cases that were manually interpreted [24,25]. Furthermore, there are no prospective randomized controlled studies comparing the accuracy of AI as a stand-alone breast cancer screening interpretation system with that of radiologists’ interpretations. In retrospective studies, AI systems were inferior to radiologists in terms of interpretation accuracy [33]. However, through DL and model training of image data, there is still great potential to use AI to complete early breast cancer screening in the future [27].

#### 3.2.1. Mammograph in Breast Cancer

Breast cancer mortality among women in the United States has continued to decline since the introduction of mammography screening. Women with breast cancer who underwent mammography screening demonstrated a 53% lower risk of breast cancer-related death compared to those with intermittent/incidental breast cancer [34]. However, radiologists usually determine the properties of a lesion based on its image characteristics on the mammogram, such as density, homogeneity, and margins, which relies on the physician’s personal perception and experience and suffers from subjective cognitive bias. Sahiner et al. first attempted to use CNN for mammogram image analysis, although only a simple model with few layers was used, mammography techniques and the application of AI in mammography image analysis have been greatly developed since then [35,36]. The application of CNN in mammography diagnosis mainly contains benign and malignant tumor identification, mass localization, segmentation of cancerous, and non-cancerous tissues, and breast classification based on density [37]. CNN algorithm in benign and malignant tumor mass identification is only accurate in the lesion with BI-RADS classification of grade 1, while it is difficult to diagnose images of higher grade tumors [38]. CNN is weak in identifying tumor lesions in dense breast and pectoral muscle when used for mass localization in mammography [39,40,41]; moreover, mammography does not show the extent of tumor well, and tumor staging is difficult even with manual interpretation using plain digital mammograms alone [42]. Furthermore, the use of CNN to differentiate between cancerous and peripheral normal tissues in the lesion area requires an algorithm that excludes microcalcifications and benign lesions for accurate identification [43]. 

To improve diagnostic accuracy, Pillai et al. performed breast cancer mammography detection using the VGG16 model and obtained a high accuracy, outperforming the classic AlexNet, GoogleNet, and EfficientNet models [44]. Studies have reported that CNNs can be used to identify “scattered density” and “heterogeneous density” in the BI-RADS breast density classification to categorize breasts to help predict breast cancer risk [45]. Kumar and colleagues performed breast cancer subtype classification using the VGGNet-based CNN, which reported an accuracy rate of 78% in differentiating Luminal A and Luminal B subtypes, and 67% in discerning all four subtypes of breast cancer including Luminal A, Luminal B, HER2-positive, and basal-like [46]. Singh et al. reported the application of a multi-class CNN architecture in predicting the breast tumor shapes in mammograms through a conditional Generative Adversarial Network (cGAN) and correlated the molecular subtypes of breast cancer with the predicted tumor shapes [47]. Another study explored a novel CAD system based on DCNN that helps to process deep feature extraction and fusion, which enhanced the accuracy of the SVM in classification [48]. Contrast-enhanced spectral mammography (CESM) is a new technique for angiography of breast tumors, based on plain digital mammography, using iodinated contrast agent diffused through the neovascularization of the breast within the tumor tissue to create iodine-enhanced images [49]. The uptake of contrast agent is higher in tumors compared to the surrounding normal breast tissue. Even for intraductal carcinoma, the contrast agent is able to visualize the tumor lesion through the endothelial incomplete tumor neovascularization due to the abnormal structure of the tumor neovascularization [50,51]. Investigators compared the ability of radiologists and SVM classifiers to identify malignant tumors in CESM images and suggested that SVM-based computer-aided CESM diagnosis could help radiologists reduce the number of false-positive results [52]. Another study evaluated the performance of neural network DL in identifying benign and malignant tumors in CESM images and confirmed that multimodal network image recognition can significantly reduce the biopsy rate of benign tumors [53]. Digital breast tomosynthesis (DBT) depends on anatomical changes in the breast caused by breast cancer to show the lesion, allowing different angles of the breast to be photographed and reconstructed to form a thin three-dimensional (3D) image of the breast, significantly improving the sensitivity of breast cancer imaging diagnosis [54], but accompanied by the drawback of low resolution of lesions in dense breasts [55]. DL-based AI has been used to automatically identify DBT and mammography, and researchers have used DBT image data to create maximum suspicion transmission (MSP) DL algorithms that outperformed manual reading in the identification of mammograms [27]. However, the dependence of CESM on iodine contrast media limits the use of this screening examination in people with renal insufficiency or iodine allergy.

#### 3.2.2. Ultrasound in Breast Cancer

Breast ultrasound is another popular imaging detection modality for breast cancer, which is widely used in breast cancer diagnosis and guided puncture biopsy due to its non-radioactive and easy operation [56]. For small occult lesions that are not calcified, ultrasound is more advantageous than mammography [57,58,59]. In addition to conventional breast ultrasound, there are ultrasound elastography, ultrasonography, automated full-volume breast scan imaging, ultrasound light scattering tomography, etc. These ultrasound-based detections integrate multiple ultrasound contrast agents, 3D imaging technology, and spectral analysis technology on the basis of general ultrasound to achieve a variety of diagnostic needs such as assessment of tumor texture, differentiation of benign and malignant, and display of the relationship with surrounding tissues. Han et al. reported the identification of benign and malignant masses using CNN for breast ultrasound images, with an accuracy of 90%, and diagnostic sensitivity and specificity of 86% and 96%, respectively [60]. The recent advent of ultrasound elastography has greatly improved the accuracy of ultrasonography in the diagnosis of breast cancer, and the semi-quantitative assessment of lesion stiffness enables a more accurate differentiation of lesion benignity and malignancy [61]. Zhang et al. used a two-layer DL algorithm model to discriminate the properties of tumor masses using elastic shear wave images, with a diagnostic accuracy of 93.4%, sensitivity of 88.6%, and specificity of 97.1% [62]. Combining the imaging features of breast ultrasound and ultrasound elastography for histological analysis can be used to preoperatively predict the status of axillary lymph node metastasis in clinical T_1-2_ breast cancer [63]. 

Compared to DL applied to CT and MRI image recognition, ultrasound examination is an operator-dependent imaging modality, and in-depth AI recognition of ultrasound images suffers a significant subjective bias in the operation of ultrasonographers and in the reading of films by ultrasound physicians. Therefore, AI recognition of ultrasound images is more dependent on communicating DL models with ultrasonographers than CT and MRI, which partly induces the development of AI recognition of ultrasound to lag behind that of CT and MRI. To overcome the disadvantages of the breast ultrasound image, Singh et al. presented a breast ultrasound segmentation method using contextual-information-aware deep adversarial learning framework, whereby the breast ultrasound image can be segmented efficiently and handle various tumors with distinct sizes and shapes [64]. Hassanien et al. presented a new DL-based radiomics method called ConvNeXt to endow the CAD to predict the malignancy score of a breast lesion. By using a vision transformer style, the ConvNeXt system is able to perform the malignant score analysis of breast ultrasound sequences and present visual interpretations for its decision [65]. Jabeen and colleagues optimized feature extraction and improved breast cancer categorization accuracy to 99.1% by modifying and retraining the deep model named DarkNet53. They achieved the best feature selection using reformed deferential evolution (RDE) and reformed gray wolf (RGW) optimization algorithms, and these features were fused and categorized with the probability-based approach and ML algorithms, respectively [66].

#### 3.2.3. MRI in Breast Cancer

Compared with the previous two imaging methods, breast MRI is the most sensitive and accurate imaging method for preoperative staging of breast cancer, and its sensitivity for tumor diagnosis is not affected by the density [11,67]. MRI provides information indicating tumor biological function: spectral imaging can determine the function of chemical components within tissue regions for qualitative tumor diagnosis through metabolite detection [68]; or MRI spectral imaging can be used for quantitative assessment of lipid composition in the breast based on altered adipogenesis genes in breast cancers with vascular infiltration, allowing prediction of tumor vascular infiltration events [69], assessment of chemotherapy response [70], and subtype identification [71]. Diffusion-weighted imaging (DWI) is highly sensitive for breast cancer as it reveals and evaluates local pathophysiological features by measuring the mobile phase of water molecules in the tissue. MRI has been reported to play a multifaceted role in the diagnosis of breast cancer, for example for pCR prediction of breast cancer after neoadjuvant therapy [72,73]; diffusion-weighted (DWI-MRI) and contrast-enhanced MRI (CE-MRI) have the advantages of high sensitivity and specificity, respectively, in neoadjuvant efficacy monitoring of breast cancer, and the combination of the two diagnostic imaging techniques is expected to improve the accuracy of neoadjuvant efficacy assessment [74]. The utilization of CE-MRI can provide four types of features: tumor morphology, texture, hemodynamic, and pharmacokinetics, among which kinetic features are a unique detection advantage of MRI over the previous two imaging examinations, which can help in tumor identifying and classification by showing hemodynamic features of tumors that are completely different from those of normal glands [75]. Some studies have reported that the accuracy of MRI for neoadjuvant efficacy evaluation of breast cancer varies between different subtypes of breast cancer, and MRI is more suitable for evaluating the neoadjuvant therapy efficacy in human epidermal receptor-2 (HER2)-positive breast cancer and triple-negative breast cancer (TNBC) subtypes compared to luminal subtype [76]. A growing trend in new application studies is multimodal image reconstruction with a combination of multiparametric MRI, i.e., combining one or several MRI techniques to provide more accurate imaging evaluation by combining the advantages of various examinations. Winkler and colleagues confirmed the feasibility of DL in identifying breast tumor lesion-containing slides in MRI images. To improve the clinical workflow of breast MRI viewing, they integrated the DL technology into the picture and archiving communication system (PACS), whereby radiologists can quickly choose the targeted image instead of scanning the imaging stack [77].

#### 3.2.4. Nuclear Medicine Techniques

The employment of nuclear medicine examination represented by ^18^F-FDG PET/CT in breast cancer covers diagnosis, staging, assessment of recurrent metastases, phenotypic identification, prognosis, and assessment of treatment response. Compared with other imaging examinations, PET has the advantage of obtaining whole-body staging information by a single examination [78,79,80]. The uptake of FDG has been shown to correlate with tumor grade and proliferation index [81]. Nuclear medicine tests, such as 18F-FDG PEM or dedicated breast PET (dbPET), have shown higher sensitivity and specificity compared to PET/CT in several studies for early breast cancer diagnosis [82]. Other studies have reported ^64^Cu-DOTA-trastuzumab PET/CT and MRI for establishing predicting model of neoadjuvant response in HER2-positive breast cancer; using ^18^F-FES PET/CT for predicting the chemotherapy efficacy of MBC [83], or using FDG PET/CT to screen candidates for sentinel lymph node biopsy or axillary lymph node dissection [84]. However, PET/CT is not suitable for detecting small intracranial metastases due to the higher glucose metabolic background of brain parenchyma, and the cost-effectiveness of this examination in early breast cancer needs further evaluation considering the high expense. MRI shows higher accuracy than other imaging examinations in breast cancer diagnosis, encouraging the application of fusion imaging of ^18^F FDG-PET/CT with MRI in breast cancer. The imaging fusion technology has further improved sensitivity and specificity for breast cancer detection compared to ^18^F FDG-PET/CT or MRI alone [79,85]. PET/MRI has better staging capability and lower radiation dose than PET/CT for breast cancer [86], and has a sensitivity of 90-99% for breast cancer detection. However, the specificity of breast cancer diagnosis is only 72-89%, which may augment the FPR, and the biopsies proportion [79], and potentially cause the problem of higher examination costs. Due to the small sample sizes, inconsistent study designs, high heterogeneity of imaging data between studies, and lack of uniform criteria such as indications and scan parameters, the evidence is not clear on the role of PET/CT and PET/MRI in breast cancer.

#### 3.2.5. Radiomics and Radiogenomics in Breast Cancer

Traditional imaging methods diagnose the properties of tumors by the qualitative characteristics of tumor lesions in the imaging images, including tumor density, intratumor components (such as tumor parenchymal components, blood vessels, necrosis, and calcification), the morphology of tumor lesion edges, and the anatomical relationship with surrounding tissues. The rapid development of sequencing technology has enabled the detailed study of genetic information in all dimensions of human diseases, and further expanded to the analysis of multiple dimensions of biological information, i.e., “multi-omics” research, such as the multi-modal integrative analysis of genomics, transcriptomics, proteomics, etc. Multi-omics research provides a more comprehensive view of the biological processes of human physiology and pathology. Along with this, the concept of radiomics has also been introduced in the field of imaging research, where AI is used to extract features from images (X-ray, CT, ultrasound, and MRI) through high throughput and enable digital decoding of radiographic images into quantitative features, establishing AI-based image feature recognition as a clinical diagnostic aid. Similar to other multi-omics, the purpose of radiomics is to study the molecular biology of a lesion through its imaging performance. The image features extracted from radiomics reflect not only their appearance on the image but also the alterations that occur at the genetic and molecular levels. 

The general process of image histology analysis is similar to that of AI-based diagnostic imaging techniques, including the acquisition of high-quality image data, outlining the region of interest (tumor segmentation), AI-based high-throughput image feature extraction, feature screening using algorithms based on the selected predictive/assessment endpoint events, model construction using the screened image features, and model validation using internal and external datasets. A DBT-based imaging histology analysis found a correlation between tumor size and estrogen receptor status [87]. A radiomics study using breast MRI images revealed a correlation between entropy and tumor vascularity and heterogeneity, which can be applied to identify benign and malignant breast tumors [88]. Another radiomics study using TCGA/TCIA public MRI data enrolled 91 cases of biopsy-proven invasive breast cancer for molecular typing by radiomics. The model developed in this study was able to better distinguish the expression status of prognostic molecular markers, including estrogen receptor, progestin receptor, and HER2. The study also observed a correlation between tumor size and tumor aggressiveness at the same time [89]. Radiomics analysis of MRI was also used for the prediction of axillary lymph node metastasis in breast cancer and further incorporated clinical features based on radiomic models to provide a nomograph for predicting the risk of axillary lymph node metastasis and recurrence in patients with early breast cancer. Although this study was also retrospective, with large heterogeneity in MRI scans and a short follow-up period, its findings provide a valuable direction for the application of MRI-based imaging radiomics in breast cancer diagnosis [90]. Yu et al. also used MRI radiomics to develop a model for preoperative predicting axillary lymph node metastasis in breast cancer. Since the metastatic axillary lymph nodes were of the same origin as the primary breast foci, the study incorporated both the primary breast foci and the lymph node metastases to develop a prediction model for axillary lymph node metastasis, which improve prediction accuracy than the prediction model developed only with the primary foci features [91]. 

ET/CT images have also been reported to allow for radiomics analysis. Poor therapeutic outcome due to tumor heterogeneity is a leading research topic in the field of drug resistance. Using 18F-FDG PET/CT-based images of locally advanced breast cancer before and after neoadjuvant therapy, the tumor heterogeneity texture parameters were extracted, and the obtained metabolomic patterns correlated with ki67 expression, pCR rate, and risk of recurrence after neoadjuvant [92]. CEM-obtained breast images combined with manual segmentation of tumor lesions can better facilitate DL-based automatic extraction of tumor features and identification of invasive and non-invasive breast cancers [93]. The study used CEM images of the breast with manual segmentation of lesion areas for radiomics analysis and achieved the identification of histological and molecular subtypes of HER2-positive and triple-negative breast cancers successfully [94]. The development of new diagnostic imaging modalities for breast cancer using radiomics is highly promising, and the accuracy and efficiency of breast cancer diagnosis can be improved by combining radiomic analysis with conventional imaging. However, the diagnostic accuracy of some of the classification models currently developed to identify benign and malignant tumors is still lower than manual identification, and more research is still needed to refine the algorithms and define indications to establish a general and stable automated analysis tool.

Correlations between medical imaging features and gene expression pathways have been shown to provide information on the constitute of lesion genetics, for example, imaging features of tumor size are positively correlated with cell proliferation-related gene expression and signaling pathways [95]. Radiogenomics perform joint analysis of radiomics and genomics to provide more informative and individualized radiological and genetic features [95], allowing the concept of precision medicine and personalized therapy to be no longer limited to studies at the genomic or proteomic level that rely on tissue or blood samples. Radiogenomics studies can help in prediction and early detection of cancer by using imaging features to analyze changes at the molecular level of the disease. Multiple different combinations of imaging genomics analysis have arisen because there are multiple dimensions of genetic testing, such as DNA sequencing and RNA sequencing, as well as different image features obtained by different imaging examinations. The study by Yeh et al. combined RNA-seq data with 3D DCE-MRI images to establish robust associations between multiple signaling pathways involving cell growth and death, immune regulation, intercellular interactions, and signal transduction with enhanced MRI images, and the up- or down-regulation of these signaling pathways were associated with the different imaging phenotypes of tumors [95]. Another advantage of imaging genomics is the possibility of obtaining comprehensive information at the molecular level of genes within the focus by resolving imaging information of the whole lesion, which cannot be acquired by puncture or small sample biopsy due to tumor heterogeneity. The Cancer Genome Atlas (TCGA) program has linked the completed genomic and clinical biomarkers for adult cancer identification with the data from The Cancer Imaging Archive (TCIA) [96,97]. Currently, radiogenomics lacks standardized processes and assessment criteria to ensure quantitative imaging, and various discrepancies between imaging scans may lead to over- or under-treatment. Thus, while radiogenomics is much more effective than histopathological imaging, the process involves very expensive and requires large sample data sets and powerful computational capabilities for validation. Radiogenomics is not routinely practiced in clinical practice.

## 4. Discussion

AI-assisted imaging diagnosis is a promising strategy in breast cancer diagnosis. Several potential directions of AI application are attractive: 1. AI-based diagnosis as minimally invasive or non-invasive tests that can satisfy the purpose of convenient efficacy assessment; 2. differentiation of distinct molecular biological features in primary or recurrent/metastatic loci; 3. obtaining information on tumor heterogeneity; 4. identification of treatment response or tumor progression (present in immunotherapy). However, the current techniques, from imaging detection methods to lesion segmentation and qualitative diagnosis of images to analysis of lesion image features, are deficient and inadequate to support the use of AI-assisted imaging diagnosis to complete independent imaging or even clinical diagnosis. Several key issues limit the prevalence of AI-assisted imaging diagnosis in breast cancer. First, there is a lack of a generally recognized operation standard of the process, ranging from imaging detection and tumor segmentation to image feature extraction, to ensure reproducible results. Secondary, novel algorithms with higher specificity are needed to cope with various kinds of images of distinct quality as well as patients’ individual variations. Third, even though imaging-based AI diagnosis can be potentially applied in breast cancer to reduce the FPR, the diagnostic accuracy relies on the image quality of imaging detections including mammography, ultrasound, etc., and the algorithm is still waiting to be refined to improve the diagnostic accuracy. Furthermore, the clinical practical value of AI-assisted breast cancer diagnosis still needs to be validated in randomized clinical trial with large perspective cohort.

## 5. Conclusions

AI-assisted imaging diagnosis provides a promising perspective of a more accurate and high-efficient diagnostic model for breast cancer. However, further optimization and validation in randomized clinical trials are essential before it is applied in clinical practice. Moreover, a breakthrough in the direct connection pathway between AI-specific training/diagnostic databases and health insurance databases or hospital information systems (HIS), can help accelerate the establishment of a robust, open, self-optimizing AI imaging platform for breast cancer diagnosis.

## Data Availability

Data sharing not applicable.
